# Scale-dependent effects of habitat area on species interaction networks: invasive species alter relationships

**DOI:** 10.1186/1472-6785-12-11

**Published:** 2012-07-20

**Authors:** Shinji Sugiura, Hisatomo Taki

**Affiliations:** 1 Department of Forest Entomology, Forestry and Forest Products Research Institute (FFPRI), 1 Matsunosato, Tsukuba, Ibaraki, 305-8687, Japan

**Keywords:** Alliaria petiolata, Biological invasion, Forest area, Mutualistic networks, Plant–pollinator interactions, Species–area relationships

## Abstract

**Background:**

The positive relationship between habitat area and species number is considered a fundamental rule in ecology. This relationship predicts that the link number of species interactions increases with habitat area, and structure is related to habitat area. Biological invasions can affect species interactions and area relationships. However, how these relationships change at different spatial scales has remained unexplored. We analysed understory plant–pollinator networks in seven temperate forest sites at 20 spatial scales (radius 120–2020 m) to clarify scale-associated relationships between forest area and plant–pollinator networks.

**Results:**

The pooled data described interactions between 18 plant (including an exotic) and 89 pollinator (including an exotic) species. The total number of species and the number of interaction links between plant and pollinator species were negatively correlated with forest area, with the highest correlation coefficient at radii of 1520 and 1620 m, respectively. These results are not concordant with the pattern predicted by species–area relationships. However, when associations with exotic species were excluded, the total number of species and the number of interaction links were positively correlated with forest area (the highest correlation coefficient at a radius of 820 m). The network structure, i.e., connectance and nestedness, was also related to forest area (the highest correlation coefficients at radii of 720–820 m), when associations with exotics were excluded. In the study area, the exotic plant species *Alliaria petiolata*, which has invaded relatively small forest patches surrounded by agricultural fields, may have supported more native pollinator species than initially expected. Therefore, this invasive plant may have altered the original relationships between forest area and plant–pollinator networks.

**Conclusions:**

Our results demonstrate scale-dependent effects of forest area on the size and structure of plant–pollinator networks. We also suggest that a single exotic plant species can impact plant–pollinator networks, even in temperate continental habitats.

## Background

The relationship between species number and island area, namely, that species number increases with increasing island area, is a fundamental rule in ecology [1-4]. MacArthur and Wilson [1] hypothesized that this relationship results from a dynamic equilibrium between opposing immigration and extinction rates, which depend on island isolation and size, respectively. Because the same trend occurs in continental environments [1-3], the theory of island biogeography has been applied to the conservation of continental habitats [5-8].

Species–area relationships suggest that the number of interaction links among species, such as prey–predator and plant–pollinator species, increases with island or habitat area [9,10]. However, surprisingly, only a few studies have tested this prediction, including one that examined plant–ant interactions on oceanic islands [9], and another that studied plant–pollinator interactions on continental habitats [11]. Additionally, the network structure of species interactions is empirically known to relate to the total number of species interacting [12-14], which raises the expectation that there may be a close relationship between area and network structure [9]; however, only one study has tested this prediction at the landscape scale [15].

Species–area relationships differ among spatial scales, with the shapes and slopes of the relationships differing among local, regional, and global scales [3,16-18]. In addition, the effects of spatial scale on species–area relationships may depend on taxonomic group; for example, responses to habitat area differ between animals and plants [3,19]. Given that networks of species interactions usually involve different taxonomic groups [20,21], different spatial scales may affect the relationships between these networks and habitat area. However, how habitat area influences interaction networks at different spatial scales at the landscape level has remained unexplored.

Biological invasions impact the interactions among native species [22,23], and island communities are more likely to be invaded and affected by exotic species than continental ones [24-26]. However, exotic species may invade continental communities disturbed by human activities [14,24]. Therefore, exotic species may impact the original relationship between habitat area and interaction networks even in continental environments.

Here we analysed plant–pollinator networks, both including and excluding exotic species, in temperate continental forests at different spatial scales to clarify scale-variable effects of habitat area on interaction networks. Plant–pollinator interactions provide excellent model systems for investigating the structure of species interactions [12,27-29]. Plant–pollinator interactions have recently been targeted for studying the network structure of plant–animal mutualistic interactions [28-30]. For example, network metrics such as connectance and nestedness are often used to clarify the structure of mutualisitic interaction networks [12-14,31,32]. As network metrics are correlated with the total number of interacting species [12-14,32,33], and the number of species is related to habitat area [1-3], we expect that network structure is also related to habitat area. Reports of such area-related effects on network structure are rare [9], although the mathematical consequences of changing species numbers for network structure have been discussed [31,34].

The main goal of this study was to test the following hypotheses: (1) the total number of species increases with forest area, (2) the network metrics of plant–pollinator interactions are related to the total number of species and forest area, (3) the relationships between forest area and plant–pollinator networks are scale-dependent, and (4) exotic species impact the relationships between forest area and plant–pollinator networks. Although the effects of island and habitat area on interaction networks have recently been reported [9,11,15], the scale-dependencies have never been examined.

## Methods

### Study system

**Figure 1:**
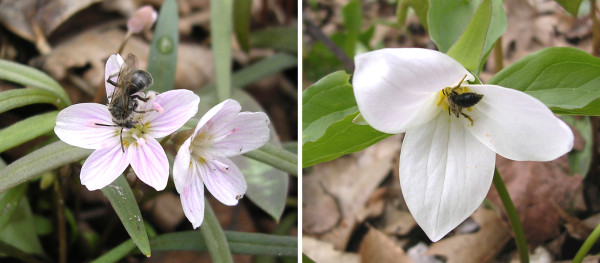
***Andrena *bee visiting a flower of *Claytonia virginica *(left) and *Trillium grandiflorum *(right) in the forest understory.**

We analysed a data set that was in part used by Taki and Kevan [35] to examine the effects of forest loss on the degree of specialization/generalization for plant and insect communities. Our analyses focused on the effects of forest area on the size and structure of the whole network in terms of scale-dependency and biological invasion. The study was conducted in Norfolk County, Ontario, Canada ( Additional file 1; 42°37'–42°48' N, 80°25'–80°39' W; ca. 200 m a.s.l.). The study forests were located in southern Ontario’s deciduous Carolinian forest zone. The landscape is flat and characterized by distributed fragments of forest within intensively managed agricultural fields of crops such as corn, soybean, and tobacco. The forests were composed of deciduous trees such as oak and maple [36]. Small shrubs and herbs dominated the forest floors. The associations of flower-visiting insects with understory plants that flowered prior to leaf flush of the forest crown trees were examined (Figure 1) by observing the interactions between 18 flowering plants and 89 flower-visiting insect species ( Additional file 2 and Additional file 3). Although it is possible that not all of the flower-visitors were legitimate pollinators, we considered them to be pollinators to decipher the general patterns between habitat area and plant–pollinator networks. Garlic mustard *Alliaria petiolata* and honeybee *Apis mellifera*, both of which originated in Europe, are exotic species in this region ( Additional file 2 and Additional file 3). Both species are widely acknowledged to impact native communities [37,38]. However, it is unlikely that *A. mellifera* has become established in the study forests, and the small numbers of individuals captured in this study likely escaped from domesticated colonies. *Alliaria petiolata*, on the other hand, has become established in these areas, and it invades forest edge and understory. 

### Study design

The study was conducted in seven forest sites [35] ( Additional file 1), which were selected using a Geographical Information System (GIS) with ArcView (Version 3.3, ESRI, Redlands, CA, USA). Seven geographical points that fell within the forest polygons in the study region were randomly chosen. The criteria for accepting a selected point included a minimum distance of 40 m from all edges of the forest polygons and a minimum separation distance of 4500 m from any other chosen geographical points. The geospatial data of forest coverage were produced using aerial photography (1:30000 and 1:50000) obtained from the Ontario Base Map Series in 2003 (Ontario Ministry of Natural Resources, Peterborough, ON, Canada).

Each of the seven selected sites consisted of a hexagonal transect with 20 m sides with the chosen geographical point marking the centre ( Additional file 4). In constructing the hexagonal transects, the direction of the first radial arm of each transect was randomly chosen using a 1.5 m stick thrown into the air. The axes were marked with bamboo poles and a 120 m section of rope demarcated the perimeter. Although a better choice for transect shape would be a circle, hexagonal transects were more practical.

A belt transect method [39] was applied to the hexagonal transects. We sampled before canopy closure because most understory plant species bloom during this short season [40-42]. Flower-visiting insects were sampled on sunny days when the temperature was at least 12.8°C. Sampling started at 11:30 and at 14:30, times at which flower visitations by insects are relatively frequent. Two sites were sampled each day in most cases. Four sampling cycles were conducted to ensure that sampling was carried out twice at 11:30 and twice at 14:30 for each of the seven sites (i.e., each site was sampled on four different days). All samplings were conducted from late April to late May. At each sampling, the same two researchers walked the perimeter of the hexagons five times at a slow pace. One person walked clockwise and the other walked counter-clockwise. Each of these samplings took 80–100 min. All insects visiting the flowers within 2 m on either side of the perimeter rope were sampled with insect nets and aspirators. The species of the flowering plants visited by each sampled insect were recorded. The species richness and abundance of all flowering plants in bloom at each site were recorded using a quadrate method with 1 x 1 m quadrates placed every meter along each side of the perimeter of each hexagon, creating 20 quadrates along the outer perimeter of each side and 19 along the inner row (234 quadrates in total for each recording). The cataloguing and census of plants took place four times for each site, coinciding with the first, second, third, and fourth insect sampling cycle (described above).

To quantify the amount of forest area at different spatial scales, circles of 20 radii from 120 to 2020 m (100–2000 m from the hexagonal transects) with 100 m intervals were created using ArcView on maps around each of the hexagonal transects. The scales followed previous studies on foraging ranges and scale-dependent effects in flower-visiting insects such as bees [43-46]. The forest area at each of the scales was estimated by the amount of forest coverage (km^2^) within the circles. The environment surrounding the forests, i.e., intensively managed annual agricultural fields, was not habitat for most of native plant or pollinator species recorded in the study forests. Therefore, we can consider the forest coverage as habitat area for most plant and pollinator species (except for honeybees).

The degree of connectivity among forest patches has been considered one of the most important factors for evaluating the impacts of forest fragmentation on biodiversity [e.g., [47]. In this study area, Taki et al. [46] showed that bee abundance and diversity increased with increasing forest area but not increasing forest edge. This suggests that connectivity has a lesser influence on plant-pollinator networks than the overall forested area in this study region. Therefore, our analyses did not focus on the effects of forest connectivity but rather the effects of forest area on plant-pollinator networks.

### Data analysis

The numbers of species and interaction links were pooled at each forest site for the analyses. To determine the impacts of exotic species on the relationships between forest area and plant–pollinator networks, two data sets were analysed; the first included exotic species (i.e., contained all species) and the second excluded associations with exotic plant and pollinator species (i.e., contained only native species).

The numbers of plant species, pollinator species, total species, and occupancy by exotic garlic mustard *A. petiolata* were analysed in relation to forest area using Pearson’s correlation coefficient at each spatial scale. Prior to the analysis, forest area and species numbers were log transformed; occupancy of flowering *A. petiolata* (proportion of maximum numbers of quadrates out of 234) was square root-arcsine transformed.

To examine how the network metrics were related to the total number of species and forest area, the number of interaction links, connectance, and nestedness were calculated. On the basis of previous studies [9,11], the number of observed interaction links between plant and pollinator species (*I*) can be predicted to increase with increases in the total number of species and habitat area.

Connectance (*C*), or the proportion of interactions actually observed amongst all possible interactions, usually represents the degree of redundancy in a system, with consequences for community stability [12,48]. It was calculated as follows [12,31,48]:

(1) C=I/(Pl×Po),

where *Pl* and *Po* are the numbers of plant and pollinator species, respectively. On the basis of previous studies [12,14,32,33], connectance can be predicted to decrease with increases in the total number of species and habitat area.

Nestedness is a pattern composed of asymmetrical interactions between generalists and specialists and symmetrical interactions among generalists. Nestedness is frequently detected within plant–pollinator networks [13,29,30,49], although the concept of nestedness was originally used to analyse patterns of species occurrence and absence on a set of islands or in habitat fragments [50]. Nested networks are characterized by specialist species that tend to interact only with generalists; generalists that all interact with each other, forming a core of interacting species; and the absence of specialists that interact only with other specialists [28,30]. We used the software ANINHADO 3.0.3 [51,52] to calculate nestedness (*NODF*; range 0–100). Relatively large values of *NODF* indicate a high degree of nestedness. Two null models (Models I and II provided by ANINHADO 3.0.3) were used to test the degree of nestedness expected from the basic network features. The first null model assumes that each randomly assigned plant and pollinator pair interacts with a constant probability, *C* (connectance). Therefore, it tests whether the observed *NODF* is higher than expected for random networks with a similar number of interactions. The second null model assumes that the probability of a plant, *i*, interacting with a pollinator, *j*, depends on the observed number of interactions of both species, such that

(2) $$C(r_{\mathrm{ij}}=1)=\left(\frac{k_{i}}{Po}+\frac{k_{j}}{Pl}\right)\frac{1}{2}$$

where *k* is the observed number of interactions for the species, *Pl* is the number of plant species (rows) and *Po* is the number of pollinator species (columns). Therefore, null model II tests whether the observed *NODF* is higher than expected for random networks with similar heterogeneities of species interactions. Each community was compared to 1000 replicates generated by each null model. On the basis of the previous study [13], nestedness in mutualistic networks is predicted to increase with increases in the total number of species and habitat area.

The network metrics, link numbers, connectance, and nestedness were also analysed in relation to forest area using Pearson’s correlation coefficient at each spatial scale (JMP v. 7.0; SAS Institute, Cary, NC, USA). Before the analysis, *C* and *NODF*/100 were square root-arcsine transformed while link numbers and forest area were log transformed. Linear regression models were used to examine the relationships between the total number of species and network metrics (JMP v. 7.0). We also used linear regression models to investigate the relationships between forest area and network metrics. As the scale for linear regressions, we used the radius at which the highest correlation coefficient was detected.

## Results

### Relationships between forest area and species numbers

**Figure 2:**
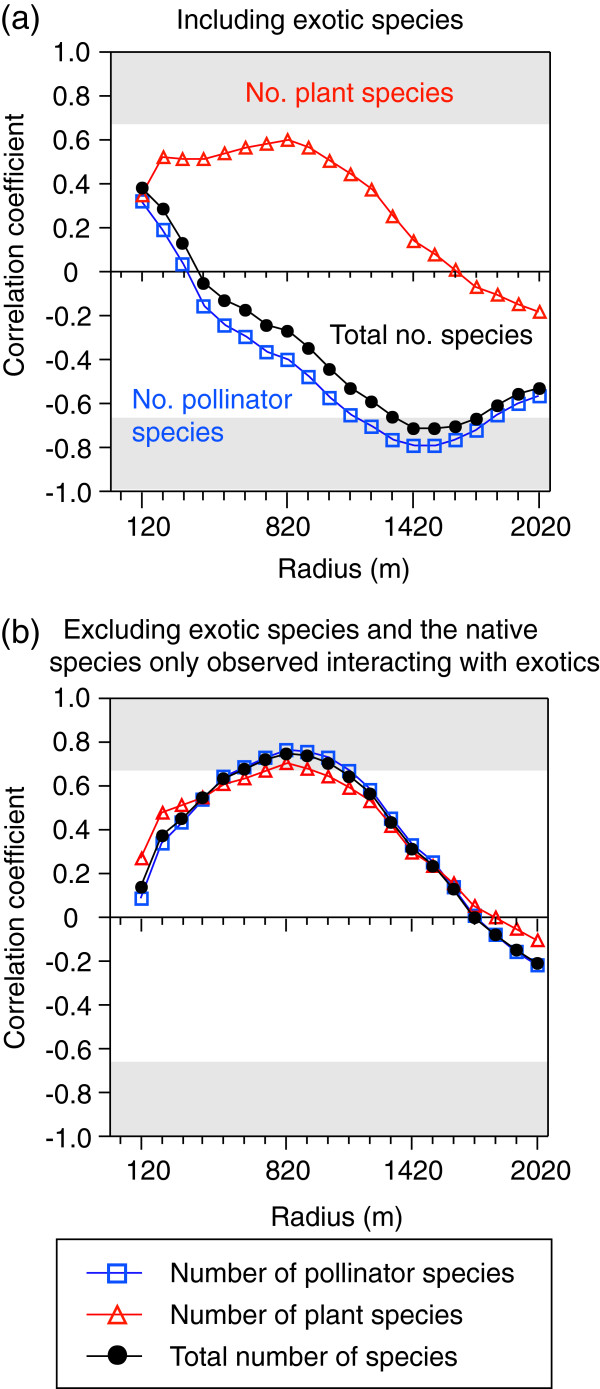
**Correlation coefficients between forest area and numbers of species at each radius (range: 120–2020 m) among the study forests: (a) including exotic species, (b) excluding exotic species and the native species only observed interacting with exotics.** Correlation coefficients in the shaded grey areas are statistically significant at *p* = 0.10 level.

**Figure 3:**
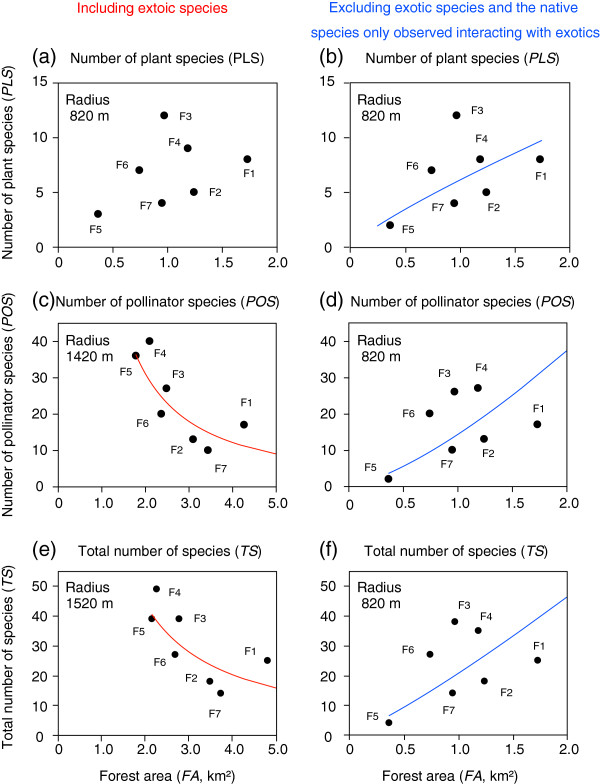
**Relationship between forest area and (a, b) number of plant species, (c, d) number of pollinator species and (e, f) total number of species.** Data for the radius with the highest correlation coefficients (Figure 2) regarding the effects of forest area are shown. Left (**a**, **c**, **e**) and right figures (**b**, **d**, **f**) are based on data including exotic species and excluding associations with exotics, respectively. Code numbers next to the circles indicate study site codes, which are provided in Table 1. Lines represent linear regressions: (**b**) number of plant species (*PLS*), log(*PLS*) = 1.8062 + 0.8348*log(*FA*), *r*^2^ = 0.49, *F*_1,5_ = 4.77, *p* = 0.081; (**c**) number of pollinator species (*POS*), log(*POS*) = 4.3847 – 1.3624*log(*FA*), *r*^2^ = 0.63, *F*_1,5_ = 8.60, *p* = 0.033; (**d**) log(*POS*) = 2.6504 + 1.3926*log(*FA*), *r*^2^ = 0.58, *F*_1,5_ = 6.88, *p* = 0.047; (**e**) total number of species (*TS*), log(*TS*) = 4.5598 – 1.1211*log(*FA*), *r*^2^ = 0.51, *F*_1,5_ = 5.29, *p* = 0.070; (f) log(*TS*) = 3.0195 + 1.1740*log(*FA*), *r*^2^ = 0.56, *F*_1,5_ = 6.30, *p* = 0.054.

**Figure 4:**
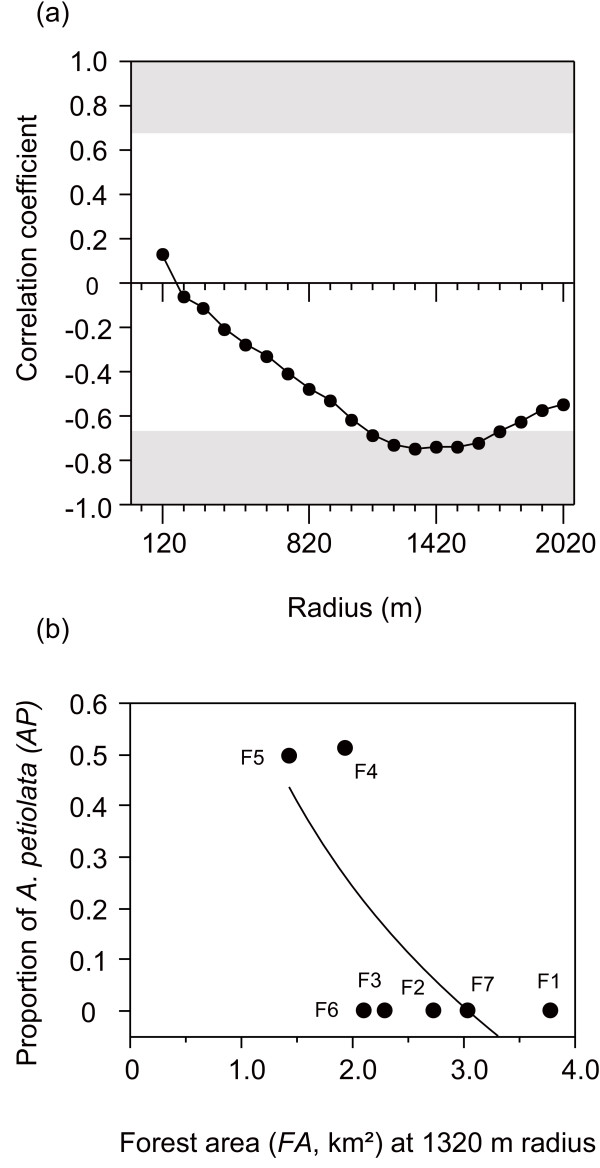
**Relationship between forest area and occupancy by the exotic plant *Alliaria petiolata.*** (**a**) Correlation coefficient between forest area and occupancy by *A. petiolata* at each radius (range: 120–2020 m) among the study forests. (**b**) Relationship between forest area at 1320 m radius and occupancy by *A. petiolatai*. Data for the radius with the highest correlation coefficients regarding the effects of forest area are shown. Code numbers next to the circles indicate study site codes, which are provided in Table 1. Correlation coefficients in the shaded grey areas are statistically significant at *p* = 0.10 level. Line represents a linear regression: occupancy by *A. petiolata* (*AP*, proportion of maximum numbers of quadrates out of 234), arcsin $\sqrt{AP}$= 0.6452 – 0.5800*log(*FA*), *r*^2^ = 0.56, *F*_1,5_ = 6.46, *p* = 0.052.

Among study sites, the numbers of plant species, pollinator species, and total species ranged from 3–12, 10–40, and 14–49, respectively (Table 1). The relationships between forest area and numbers of species were dependent on the spatial scale, and differed among plant and pollinator species (Figure 2a). The number of plant species was not related to forest area (at a radius of 820 m with the highest correlation coefficient; *r*^2^ = 0.36, *F*_1,5_ = 2.75, *p* = 0.158; Figures 2a, 3a), while the number of pollinator species and total species were negatively correlated with forest area, with the highest correlation coefficient at radii of 1420 and 1520 m, respectively (Figure 2a, 3c, 3e). These results are not concordant with the patterns predicted by species–area relationships. Occupancy by exotic *A. petiolata* was negatively correlated with forest area, with the highest correlation coefficient at a radius of 1320 m (Figure 4). When exotic species, and the native species only observed interacting with exotics, were excluded, the numbers of plant species, pollinator species and total species were positively correlated with forest area, with the highest correlation coefficient at a radius of 820 m (Figures 2b, 3b, 3d, 3f).

**Table 1:** Number of species and network metrics of plant–pollinator interactions in each study forest

**Site code**	**No. plant species**^**§**^	**No. pollinator species**^**§**^	**No. total species**^**§**^	**No. links**^**§**^	**No. visitors**^**§**^	**Connectance**^**£**^	**NODF**^**£**^
F1	8 (8)	17 (17)	25 (25)	20 (20)	34 (34)	0.147 (0.147)	11.89 (11.89)
F2	5 (5)	13 (13)	18 (18)	16 (16)	26 (26)	0.246 (0.246)	28.79 (28.79)
F3	12 (12)	27 (26)	39 (38)	34 (32)	61 (58)	0.105 (0.103)	6.83 (6.44)
F4	9 (8)	40 (40)	49 (48)	62 (40)	135 (92)	0.172 (0.185)	24.16* (17.64)
F5	3 (2)	36 (35)	39 (37)	37 (2)	94 (2)	0.343 (0.500)	5.53 (0)
F6	7 (7)	20 (20)	27 (27)	22 (22)	30 (30)	0.157 (0.157)	4.74 (4.74)
F7	4 (4)	10 (10)	14 (14)	11 (11)	14 (14)	0.275 (0.275)	14.38 (14.38)

^§^ The numbers in parentheses indicate the numbers of native plant and native pollinator species, interaction links between native plants and native pollinators, and individuals of native pollinators visiting native plants.

^£^ The numbers in parentheses indicate connectance and NODF calculated from the native plant–pollinator interactions (i.e., excluding associations with exotic species).

* *P* < 0.05 (Model I); other NODF values, *P* > 0.05 (Model I and/or II).

### Relationships between species numbers and interaction networks

**Figure 5:**
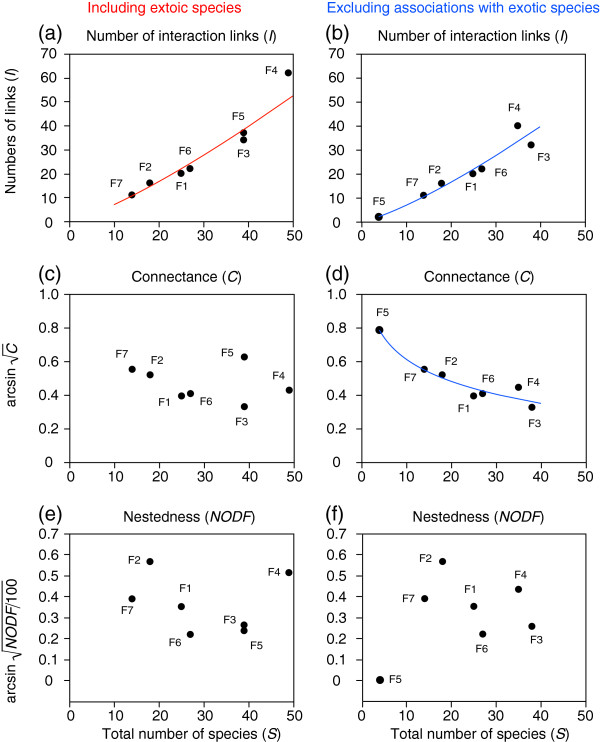
**Relationship between total number of species and network metrics for (a, b) number of interaction links, (c, d) connectance, and (e, f) nestedness. Left (a, c, e) and right (b, d, f) panels are based on data including exotic species and excluding associations with exotics, respectively.** Code numbers next to the circles indicate study site codes, which are provided in Table 1. Lines represent linear regressions: (**a**) number of interaction links (*I*), log(*I*) = −0.9601 + 1.2570*log(*S*), *r*^2^ = 0.96, *F*_1,5_ = 118.52, *p* = 0.0001; (**b**) log(*I*) = −1.0341 + 1.2774*log(*S*), *r*^2^ = 0.99, *F*_1,5_ = 334.95, *p* < 0.0001; (**d**) arcsin $\sqrt{C}$= 1.0479 – 0.1894*log(*S*), *r*^2^ = 0.94, *F*_1,5_ = 74.16, *p* = 0.0003.

The numbers of interaction links between plant and pollinator species ranged from 11–62 among the study sites (Table 1). Of these, the percentage involving exotic species ranged from 0–94.6% among the study sites (Table 1). The numbers of links were positively correlated with the total number of species (Figure 5a). This pattern did not change when associations with exotic species were excluded from the analysis (Figure 5b). Furthermore, connectance was not related to the total number of species when exotics were included (*C*, including exotics, 0.105–0.343; *r*^2^ = 0.10, *F*_1,5_ = 0.53, *p* = 0.50; Figure 5c). However, when associations with exotic species were excluded from the analysis, connectance was negatively correlated with the total number of species (*C*, excluding exotics, 0.103–0.500; Figure 5d). Most of the plant–pollinator networks were not nested (*NODF*, including exotics, 4.74–28.79; excluding exotics, 0–28.79; Table 1). The degree of nestedness was not related to the total number of species regardless of whether the associations with exotic species were excluded from the analysis (including exotics, *r*^2^ = 0.06, *F*_1,5_ = 0.33, *p* = 0.59; excluding exotics, *r*^2^ = 0.31, *F*_1,5_ = 2.21, *p* = 0.20; Figures 5e, 5f).

### Relationships between forest area and interaction networks

**Figure 6:**
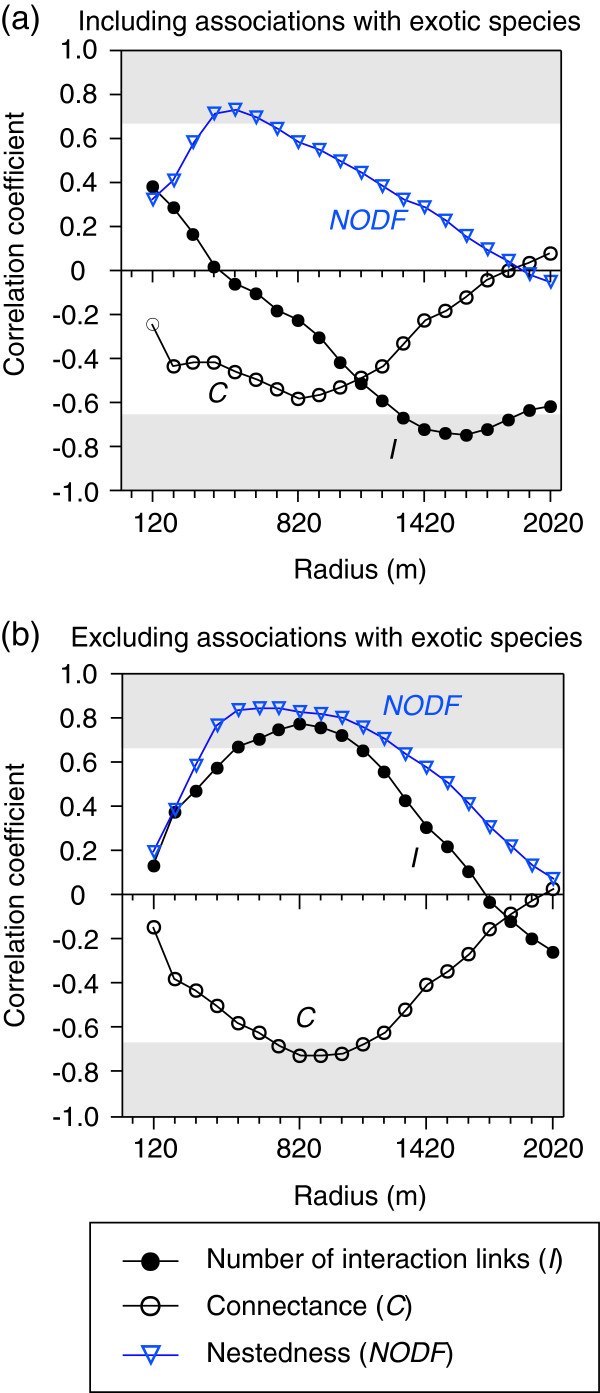
**Correlation coefficients between forest area and network metrics (number of interaction links, connectance, and nestedness) at each radius (range: 120–2020 m) among the study forests: (a) including exotic species, (b) excluding associations with exotic species.** Correlation coefficients in the shaded grey areas are statistically significant at *p* = 0.10 level.

**Figure 7:**
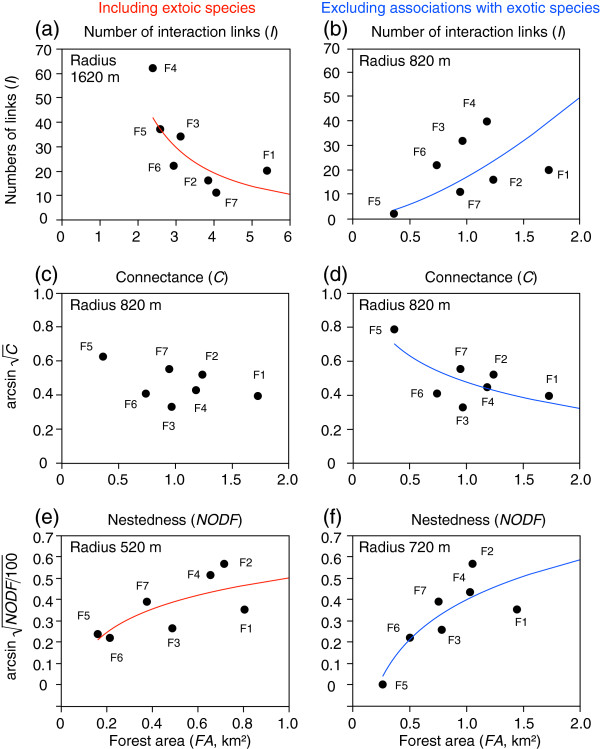
**Relationship between forest area and network metrics for (a, b) number of interaction links, (c, d) connectance and (e, f) nestedness.** Data for the radius with the highest correlation coefficients (Figure 6) regarding the effects of forest area are shown. Left (**a, c, e**) and right (**b, d, f**) figures are based on data including exotic species and excluding associations with exotics, respectively. Code numbers next to the circles indicate study site codes, which are provided in Table 1. Lines represent linear regressions: (**a**) number of interaction links (*I*), log(*I*) = 5.0660 – 1.52333*log(*FA*), *r*^2^ = 0.56, *F*_1,5_ = 6.32, *p* = 0.054; (**b**); log(*I*) = 2.8264 + 1.5556*log(*FA*), *r*^2^ = 0.59, *F*_1,5_ = 7.23, *p* = 0.043; (**c**) connectance (*C*), arcsin $\sqrt{C}$= 0.4574 – 0.1231*log(*FA*), *r*^2^ = 0.34, *F*_1,5_ = 2.59, *p* = 0.169; (**d**) arcsin $\sqrt{C}$= 0.4758 – 0.2245*log(*FA*), *r*^2^ = 0.53, *F*_1,5_ = 5.69, *p* = 0.063; (**e**) nestedness (*NODF*), arcsin $\sqrt{NODF/100}$= 0.5002 + 0.5195*log(*FA*), *r*^2^ = 0.53, *F*_1,5_ = 5.57, *p* = 0.065; (**f**), arcsin $\sqrt{NODF/100}$= 0.3963 + 0.2718*log(*FA*), *r*^2^ = 0.71, *F*_1,5_ = 12.1, *p* = 0.018.

The numbers of interaction links were negatively correlated with forest area, with the highest correlation coefficient at a radius of 1620 m (Figures 6a, 7a), which is not concordant with the patterns predicted by species–area relationships. However, when associations with exotic species were excluded from the analysis, the number of interaction links was positively correlated with forest area, with the highest correlation coefficient at a radius of 820 m (Figures 6b, 7b). Connectance was not related with forest area (at a radius of 820 m with the highest correlation coefficient; *r*^2^ = 0.34, *F*_1,5_ = 2.59, *p* = 0.169; Figures 6a, 7c). However, when associations with exotics were excluded from the analysis, connectance was negatively correlated with forest area (highest correlation coefficient at the radius of 820 m; Table 1, Figures 6b, 7d). Nestedness was positively correlated with forest area regardless of whether the associations with exotic species were excluded from the analysis, with the highest correlation coefficients at radii of 520 m including exotics and 620–720 m excluding exotics (Table 1, Figures 6, 7e, 7f).

## Discussion

Some of our results did not support our hypotheses when exotic species were included (Figures 3e, 5c,e, 6a, 7a,c). However, almost all of the hypotheses were verified when associations with exotic species were excluded from the networks (Figures 3f, 5b,d, 6b, 7b,d,f). In particular, we determined that the relationship between forest area and plant–pollinator network depended on spatial scale (Figures 2, 6). To the best of our knowledge, this is the first study to demonstrate scale-dependent effects of habitat area and exotic species on interaction networks.

### Scale-dependent effects of forest area on plant–pollinator networks

The species–area relationships were vastly different between plant and pollinator species when exotic species were included in the analyses (Figures 3a,c). Additionally, the spatial scale at which the highest correlation coefficient was found differed between plant and pollinator species (Figure 2a). Responses to habitat area are generally different between plant and pollinator species [19]. However, the scale-dependent relationship was similar among the number of plant species, pollinator species, and interaction links, when exotic species as well as native species only observed interacting with exotics were excluded (Figure 2b 6b). The similarities among the relationships between native plant and pollinator species are not likely to have been caused by our sampling scheme (i.e., insect collection on plants), because the results using a different sampling method (pan traps) also showed that native bee abundance and diversity increased with increasing forest area (with the highest correlation coefficients at 500–750 m radius [46]). The study [46] was conducted in the same region, but in different study forests (where *A. petiolata* did not flower). Therefore, the similar responses of native plant and native pollinator species to forest area caused a positive relationship between forest area and the numbers of links at the same spatial scale (i.e., 820 m; Figures 6b 7b).

Previous studies have indicated that network metrics, connectance and nestedness are related to the total number of species involved in mutualistic interactions [12-14,32,33]. Our results partly supported this pattern (Figures 5a,b,d; but Figure 5c,e,f). Although previous studies have used data sets composed of mutualistic networks at different sampling areas as well as different geographical regions [12-14,32,33], all of our data were collected from within the same sampling area (234 m^2^) in the same region. Because the total number of species was related to the forest area (Figures 3e,f), so was the network structure (Figures 7a,b,d,e,f). More redundant networks with highly asymmetric interactions were found in relatively large forest areas (Figure 7d,e,f), suggesting that the stability of plant–pollinator networks might increase with forest area. However, these relationships may partly be explained by non-biological factors. For example, connectance may decrease with increasing numbers of possible interaction links, as the number of observations per species declines when the same absolute effort is made to sample networks of different sizes [31,34]. In this study, however, connectance did not decrease with the total number of species when exotic species were included (Figure 5c). Also, nestedness was not related to the total number of species (Figures 5e,f), although it increased with increasing forest area (Figures 7e,f). These results suggest that the effects of forest area on connectance or nestedness were caused not only by changes in the total number of species, but also by other factors. For example, changes in the population densities of some species might affect the structure of interaction networks relative to habitat area because the population densities of various species are known to be related to habitat area [53]. Further studies are needed to clarify the mechanism driving the relationships between habitat area and network structure.

### Impacts of exotic species

The scale-dependent relationships among forest area, total number of species, and interaction networks were different when interactions were considered with and without exotic species (Figures 2,3,5,6,7), suggesting impacts of exotic species. Plant–pollinator interactions included only two exotic species, garlic mustard *A. petiolata* and the honeybee *A. mellifera* (Additional file 2 and Additional file 3). Although the contribution of *A. mellifera* to the interactions was not insignificant (Additional file A3), *A. petiolata* was central to the interactions in at least two forests ( Additional file 2). Although exotic plants are only rarely thought to invade temperate natural forests [24,54], *A. petiolata* has frequently been reported to invade the forest edge and understory in North America [38]. Flowers of *A. petiolata* produce rich nectar, attracting a variety of native bees and flies [55]. Nectar-rich flowers of invasive plants can disturb native plant–pollinator interactions [56]. In the study areas of the present study, *A. petiolata*, which has invaded relatively small forest patches surrounded by agricultural fields (Figure 4b), may have supported more native pollinator species than initially expected ( Additional file 3).

The presence of exotic species strongly influenced the scale-dependent relationships between forest area and the number of interaction links and connectance (Figures 7a–d). Thus, this invasive plant may have altered the original relationships between forest area and plant–pollinator networks and their scale-dependency. However, nestedness showed the same trend for both networks, with and without exotics (Figures 7e,f). Vilá et al. [23] hypothesised that invasive plants by being supergeneralists, both interacting with generalists and specialists, would increase the nestedness of the plant–pollinator network. Our results showed that excluding associations with exotic species increased the values of nestedness in some study sites (Figures 7e,f). However, this increase did not change the relationships between forest area and nestedness (Figures 7e,f).

## Conclusions

Since the original publication of the equilibrium theory of island biogeography [1], species–area relationships have been extensively studied for various groups of organisms [3,4]. Furthermore, the theory has been applied to the conservation of focal species in continental habitats [4,5]. Sugiura [9] analysed the relationships between island area and plant–ant mutualistic interactions and suggested an extension of this basic species–area relationship to more specifically address “species interactions–area relationships”. Interaction networks on continental habitats as well as oceanic islands can be considered with a view to the species interactions–area relationships. Indeed, Sabatino et al. [11] indicated that the number of interaction links among flowering plants and their pollinators increases with habitat area in the continental environment. Valladares et al. [15] also indicated that the network structure of plant–leafminer–parasitoid webs is related to fragmented forest area. In this study, we determined that network metrics of plant–pollinator interactions are related to forest area and the relationships depend on spatial scale.

Each species has a particular spatial scale of habitat area that most strongly affects the abundance because body size and mobility differ among species [19,44]. Therefore, the spatial scales of habitat area that most strongly affected the abundance generally differed among species [19]. In this study, we determined the spatial scales that most strongly affected the structure of plant–pollinator interactions, which suggests the presence of a spatial scale that most strongly influences the structuring and maintenance of the species interaction network.

Finally, exotic species appear to alter the relationship between habitat area and interaction network. Although many researchers have reported that exotic species impact interaction networks, particularly on oceanic islands [9,14,57-59], we suggest that a single exotic plant species can impact this relationship, even in temperate continental habitats.

## Competing interests

The authors declare that they have no competing interests.

## Authors’ contributions

SS and HT generated the original idea. HT designed the research. HT collected field data. SS and HT analysed data. SS wrote the paper. All authors read and approved the final manuscript.

## Supplementary Material

Additional file 1 **Seven study sites with 20 radii ranging from 120 m to 2020 m (100-2000 m from the hexagonal transects) used to obtain forest area.** All sites were in Norfolk Country, Ontario, Canada. Shaded areas represent forests, and the bar indicates a 2000 m scale. The geospatial data were obtained from the Ontario Base Map Series in 2003 (Ontario Ministry of Natural Resources, Peterborough, Ontario, Canada), Code numbers indicate study site codes, which are provided in Table 1. Click here for file

Additional file 2 List of plant species at each forest site. Click here for file

Additional file 3 List of pollinator species at each forest site. Click here for file

Additional file 4 **Hexagonal transect with 20 m sides, with the chosen geographical point marking the centre (top).** The axes were marked with bamboo poles and a 120 m section of rope demarcated the perimeter (bottom). Click here for file
