# Supplementary material for: Scale-dependent effects of habitat area on species interaction networks: invasive species alter relationships
Source: BMC Ecol. 2012 Jul 20;12:11. doi: 10.1186/1472-6785-12-11 (PMC3521176; doi:10.1186/1472-6785-12-11)
Supplement: Additional file 2 — List of plant species at each forest site. [file 1472-6785-12-11-S2.doc]

| Table S1. List of plant species at each forest site. | | | | | | | | | | | | |
| --- | --- | --- | --- | --- | --- | --- | --- | --- | --- | --- | --- | --- |
| Family | Plant species |  |  |  | Number of pollinator species | | | |  | |  |  |
|  |  | Forest 1 | Forest 2 | Forest 3 | | Forest 4 | Forest 5 | Forest 6 | | Forest 7 | | |
| Araceae | *Arisaema triphyllum* | 1 | – | 3 | | 7 | – | 3 | | – | | |
| Brassicaceae | *Alliaria petiolata** | – | – | – | | 22 | 35 | – | | – | | |
| Geraniaceae | *Geranium maculatum* | 2 | – | 8 | | – | – | – | | 6 | | |
| Liliaceae | *Erythronium americanum* | – | 1 | – | | 4 | – | 5 | | – | | |
|  | *Maianthemum canadense* | 2 | 1 | – | | – | – | – | | 3 | | |
|  | *Polygonatum pubescens* | 2 | – | 4 | | 1 | – | 4 | | 1 | | |
|  | *Smilacina racemosa* | 1 | 3 | 1 | | – | 1 | 1 | | – | | |
|  | *Trillium erectum* | 2 | – | 1 | | 1 | – | 3 | | – | | |
|  | *Trillium grandiflorum* | 8 | 10 | 4 | | – | – | 5 | | 1 | | |
| Papaveraceae | *Sanguinaria canadensis* | – | – | – | | – | – | 1 | | – | | |
| Portulacaceae | *Claytonia virginica* | 2 | 1 | – | | 8 | – | – | | – | | |
| Ranunculaceae | *Anemone quinquefolia* | – | – | 3 | | – | – | – | | – | | |
|  | *Ranunculus abortivus* | – | – | 1 | | 4 | – | – | | – | | |
| Rosaceae | *Rubus pubescens* | – | – | 1 | | 6 | – | – | | – | | |
| Rubiaceae | *Galium aparine* | – | – | – | | – | 1 | – | | – | | |
| Violaceae | *Viola labradorica* | – | – | 1 | | – | – | – | | – | | |
|  | *Viola pubescens* | – | – | 6 | | 9 | – | – | | – | | |
|  | *Viola septentrionalis* | – | – | 1 | | – | – | – | | – | | |

*Exotic species.

– no record.
