# Supplementary material for: Scale-dependent effects of habitat area on species interaction networks: invasive species alter relationships
Source: BMC Ecol. 2012 Jul 20;12:11. doi: 10.1186/1472-6785-12-11 (PMC3521176; doi:10.1186/1472-6785-12-11)
Supplement: Additional file 3 — List of pollinator species at each forest site. [file 1472-6785-12-11-S3.doc]

| Table S2. List of pollinator species at each forest site. | | | | | | | | | | | | |
| --- | --- | --- | --- | --- | --- | --- | --- | --- | --- | --- | --- | --- |
| Order | |  |  |  |  | Number of plant species | | | |  |  |  |
|  | Family | Pollinator species | Forest 1 | Forest 2 | Forest 3 | | Forest 4 | Forest 5 | Forest 6 | | | Forest 7 |
| Coleoptera | |  |  |  |  | |  |  |  | | |  |
|  | Byturidae | *Byturus unicolor* | – | – | 2 | | 1 | – | – | | | 1 |
|  | Cantharidae | *Rhagonycha* sp. 1 | 1 | – | – | | – | – | – | | | – |
|  |  | *Silis* sp. 1 | – | 1 | – | | – | – | – | | | – |
|  | Cerambycidae | *Analeptura lineola* | – | 1 | – | | – | – | – | | | – |
|  |  | *Pidonia ruficollis* | 1 | 1 | – | | – | – | – | | | – |
|  | Curculionidae | *Phyllobius oblongus* | – | – | 3 | | – | – | 1 | | | – |
|  | Nitidulidae | *Boreades abdominalis* | – | – | 1 | | – | – | – | | | – |
|  |  | *Meligethes nigrescens* | – | 1 | 1 | | – | 1 | 1 | | | – |
|  | Oedemeridae | *Oxycopis thoracica* | – | 2 | – | | – | – | 1 | | | – |
|  | Pyrochroidae | *Pedilus lugubris* | – | – | 2 | | – | – | – | | | – |
|  | Scarabaeidae | *Hoplia trifasciata* | – | 1 | – | | – | – | 1 | | | – |
| Diptera | |  |  |  |  | |  |  |  | | |  |
|  | Anthomyiidae | *Delia* sp. 1 | – | – | 1 | | 3 | – | – | | | – |
|  |  | *Delia* sp. 2 | – | – | – | | 1 | – | – | | | – |
|  | Chironomidae | Chironomid sp. 1 | 1 | – | – | | – | – | – | | | – |
|  | Mycetophilidae | *Anatella* sp. 1 | – | – | – | | 1 | – | – | | | – |
|  |  | *Docosia* sp. 1 | 1 | – | 1 | | – | – | 1 | | | – |
|  |  | *Mycetophila* sp. 1 | – | – | 1 | | 1 | – | – | | | – |
|  |  | *Mycetophilidae* sp. 5 | – | – | – | | 1 | – | – | | | – |
|  |  | *Phronia* sp. 1 | – | – | – | | 1 | – | – | | | – |
|  | Phoridae | *Megaselia* sp. 1 | – | – | – | | – | – | 1 | | | – |
|  | Rhagionidae | *Rhagio mystaceus* | – | – | – | | – | – | – | | | 1 |
|  |  | *Rhagio plumbeus* | – | – | – | | – | – | 1 | | | – |
|  | Sciaridae | *Sciara* sp. 1 | – | – | – | | 1 | – | – | | | – |
|  |  | Sciarid sp. 2 | – | – | – | | 1 | – | – | | | – |
|  |  | Sciarid sp. 3 | – | – | – | | – | – | 1 | | | – |
|  |  | Sciarid sp. 4 | – | – | – | | – | – | 1 | | | – |
|  |  | Sciarid sp. 5 | 1 | – | – | | – | – | – | | | – |
|  | Syrphidae | *Brachyopa ferruginea* | – | – | – | | 1 | – | – | | | – |
|  |  | *Brachypalpus oarus* | 1 | – | – | | – | – | – | | | – |
|  |  | *Criorhina* sp. 1 | – | – | – | | – | 1 | – | | | – |
|  |  | *Helophilus fasciatus* | – | – | – | | 2 | 1 | – | | | – |
|  |  | *Melanostoma mellinum* | – | – | 2 | | – | – | – | | | – |
|  |  | *Platycheirus* sp. 1 | – | – | – | | 1 | – | – | | | – |
|  |  | *Shaerophoria* sp. 1 | – | – | – | | – | 1 | – | | | – |
|  |  | *Syrphus rectus* | – | – | – | | 1 | – | – | | | – |
|  |  | *Syrphus ribesii* | – | – | 1 | | 2 | 1 | – | | | – |
|  |  | *Toxomerus geminatus* | 2 | – | – | | 1 | 1 | – | | | – |
|  | Tachinidae | *Epalpus* sp. 1 | – | – | – | | – | 1 | – | | | – |
|  |  | *Gonia* sp. 1 | – | – | – | | – | 1 | – | | | – |
|  |  | *Siphona* sp. 1 | – | – | 1 | | – | – | – | | | – |
| Hymenoptera | |  |  |  |  | |  |  |  | | |  |
|  | Andrenidae | *Andrena carlini* | – | 2 | 1 | | 4 | 1 | 1 | | | – |
|  |  | *Andrena crataegi* | – | – | – | | 1 | 1 | – | | | – |
|  |  | *Andrena distans* | – | – | 1 | | – | – | – | | | 1 |
|  |  | *Andrena erigeniae* | – | – | – | | 2 | – | – | | | – |
|  |  | *Andrena erythronii* | – | – | – | | 2 | – | – | | | – |
|  |  | *Andrena milwaukeensis* | 1 | – | – | | 1 | – | – | | | – |
|  |  | *Andrena nivalis* | – | – | – | | 1 | – | – | | | – |
|  |  | *Andrena rufosignata* | – | – | – | | 1 | – | – | | | – |
|  |  | *Andrena rugosa* | – | – | – | | – | 1 | – | | | – |
|  |  | *Andrena* sp. 1 (*heraclei* ?) | – | – | – | | – | 1 | – | | | – |
|  |  | *Andrena spiraeana* | – | – | 1 | | – | – | – | | | – |
|  |  | *Andrena tridens* | – | – | – | | 3 | – | – | | | – |
|  |  | *Andrena vicina* | 1 | – | 1 | | 1 | 1 | 1 | | | – |
|  | Apidae | *Apis mellifera** | – | – | 2 | | – | 1 | – | | | – |
|  |  | *Bombus bimaculatus* | 1 | 1 | – | | – | 1 | 1 | | | 1 |
|  |  | *Bombus impatiens* | 1 | – | 2 | | – | – | – | | | – |
|  |  | *Bombus perplexus* | 1 | – | 1 | | – | – | 1 | | | – |
|  |  | *Bombus vagans vagans* | – | – | 1 | | 1 | – | 1 | | | – |
|  |  | *Ceratina calcarata* | – | – | – | | 1 | 1 | 3 | | | 2 |
|  |  | *Nomada cressonii* | – | – | – | | – | 1 | – | | | 1 |
|  |  | *Nomada lepida* | – | – | – | | 1 | – | – | | | – |
|  |  | *Nomada maculata* | 2 | – | – | | – | 1 | 1 | | | – |
|  |  | *Nomada ovata* | – | – | – | | – | 1 | – | | | – |
|  |  | *Nomada sayi* | – | – | – | | – | 1 | – | | | – |
|  | Eulophidae | Eulophid sp. 1 | – | – | – | | – | – | 1 | | | – |
|  | Halictidae | *Agapostemon sericeus* | – | – | – | | 2 | 1 | – | | | – |
|  |  | *Augochlora pura pura* | – | 1 | – | | 1 | 2 | – | | | 1 |
|  |  | *Dialictus coeruleus* | – | 1 | – | | 1 | 1 | – | | | – |
|  |  | *Dialictus creberrimus* | – | – | 1 | | 2 | – | – | | | – |
|  |  | *Dialictus cressonii* | 2 | 2 | 1 | | 3 | 1 | – | | | 1 |
|  |  | *Dialictus laevissimus* | – | – | – | | – | 1 | – | | | – |
|  |  | *Dialictus nigro-viridus* | – | – | 1 | | 1 | 1 | – | | | – |
|  |  | *Dialictus pilosus pilosus* | – | – | – | | – | 1 | – | | | – |
|  |  | *Dialictus* sp. 1 | – | – | – | | – | 1 | – | | | – |
|  |  | *Dialictus* sp. 2 | – | 1 | – | | – | – | – | | | – |
|  |  | *Dialictus versans* | 1 | – | 1 | | 3 | 1 | 1 | | | – |
|  |  | *Evylaeus foxii* | – | – | 1 | | 2 | – | 1 | | | – |
|  |  | *Evylaeus quebecensis* | – | 1 | 1 | | 4 | 1 | – | | | 1 |
|  |  | *Halictus confusus* | – | – | 1 | | 2 | 1 | – | | | – |
|  |  | *Halictus rubicundus* | – | – | – | | – | 1 | – | | | – |
|  |  | *Lasioglossum coriaceum* | 1 | – | – | | 1 | 1 | – | | | – |
|  |  | *Lasioglossum parafobesii* | – | – | – | | – | – | 1 | | | – |
|  |  | *Sphecodes ranunculi* | – | – | 1 | | 1 | – | – | | | – |
|  | Megachilidae | *Osmia atriventris* | – | – | – | | – | – | – | | | 1 |
|  |  | *Osmia caerulescens* | – | – | – | | – | 1 | – | | | – |
|  |  | *Osmia sandhouseae* | 1 | – | – | | – | – | – | | | – |
|  | Pompilidae | *Priocnemis minorata* | – | – | – | | – | 1 | – | | | – |
|  | Vespidae | *Dolichovespula arenaria* | – | – | – | | 1 | – | – | | | – |
| Lepidoptera | |  |  |  |  | |  |  |  | | |  |
|  | Lycaenidae | *Celastrina ladon* | – | – | – | | – | 1 | – | | | – |

*Exotic species.

– no record.
